# Mast cell leukemia associated with undefined morphology and chronic basophilic leukemia

**DOI:** 10.1186/2052-1839-14-17

**Published:** 2014-09-13

**Authors:** Cavit Cehreli, Inci Alacacioglu, Ozden Piskin, Halil Ates, Ruksan Cehreli, Gizem Calibasi, Erdinc Yuksel, Sermin Ozkal, Guner H Ozsan

**Affiliations:** Division of Hematology, Dokuz Eylul University School of Medicine, 35330 Inciralti, Izmir, Turkey; Institute of Oncology, Dokuz Eylul University School of Medicine, Izmir, Turkey; Department of Basic Oncology, Dokuz Eylul University Institute of Oncology, İzmir, Turkey; Departments of Medical Biology and Genetics, Dokuz Eylul University School of Medicine, Izmir, Turkey; Institute of Pathology Dokuz Eylul University School of Medicine, Izmir, Turkey

**Keywords:** Mast cell leukemia, Mastocytosis, Basophilia, Interleukin-6, Dysplasia

## Abstract

**Background:**

Mast cell leukemia (MCL) is rare type of neoplasia with an incidence of 1% in a large series of 342 adult patients with systemic mastocytosis (SM). Chronic basophilic leukemia (CBL) is an extremely rare type of leukemia with appearance of 7 cases in the literature.

**Case presentation:**

A 73 year-old female patient who presented with weaknes, had a prolonged duration of hematologic remission after treatment of her CBL by hydroxyurea (HU). Evolution of SM occurring as a second neoplasia concurrently with relapse of de novo CBL was demonstrated by mast cells (MCs) infiltration in the bone marrow (BM) biopsy and smear and increase in tryptase level. Transformation to MCL with simultaneous occurrance of accelerated phase of CBL were documented by the appearance of MCs in both BM and peripheral blood (PB) smears, antigen expressions detected by flow cytometry and spesific stains. Sequence analysis of c-kit gene revealed c-kit exon 11 K550N mutation. Undefined associations of MCL with different mast cell morphology, increase in IL-6 level and accelerated phase of de novo CBL was described.

**Conclusion:**

Elevations in CRP and IL-6 levels occurring with increases in basophil counts to high levels revealed that febrile episodes with abdominal pain seen in our patient were induced by increase in IL-6 levels released from neoplastic basophils**.** Neoplastic basophils with diffuse and coarse basophilic granules possibly mimic neutrophils with toxic granules and cause wrong characterization of neoplastic basophils as neutrophils by the automated blood cell counters and misleaded physicians.

## Background

Mast cell leukemia (MCL) is rare type of neoplasia with an incidence of 1% in a large series of 342 adult patients with systemic mastocytosis (SM) [1] and accounting for <1% of all mastocytosis in the French Referance Center for Mastocytosis (CEREMAST) [2]. SM associated with clonal hematologic non-mast cell disease (SM-AHNMD) was the second most common SM subgroup (N = 138, 40%) in this cohort of patients [1]. Of The SM-AHNMD group, 89% had an associated myeloid malignancy group. This group included subgroups of, SM-myeloproliferative neoplasia (SM-MPN), SM-chronic myelomonocytic leukemia (SM-CMML) and SM-myelodysplastic syndrome (SM-MDS). A significant proportion exhibited prominent eosinophilia (>1.5 × 10^9^/l), especially those with SM-MPN and 39% harbored the *FIP1L1-PDGFRA* fusion [1, 3]. A literature search was performed by using PubMed database for all proven MCL cases according to WHO criteria and 51 adult patients with MCL were detected appearing as de novo, (n = 30) and secondary, (n = 11). The median ages for de novo and secondary cases were 51.5 (18–78) and 35.0 (5–75) respectively. Median survival was 6 months (0.5-98) in all adult patients seen in the literature [2].

CBL is an extremely rare type of leukemia with the appearance of seven cases in the literature [4–7]. Four of the seven patients reported by Pardanani et al. identified by screening of electronic data base in Mayo Clinic [4] MCL, occuring as a second neoplasia in association with undefined MC morphology and de novo CBL relapse have not been described in the World’s literature [4–7]. A case of MCL occuring as a second neoplasia in association with undefined MC morphology, increase in IL-6 levels and accelerated phase of de novo CBL was described.

## Case presentation

A 73 year old Turkish female patient who presented with weakness, epigastric fullness and decreased appetite had a prolonged duration of hematologic remission by the treatment of her CBL with hydroxyurea (HU) [7]. *At presentation*, complete blood counts (CBC) showed marked decrease in hemoglobin. Differential count made by automated blood cell counter revealed 30% segmented neutrophils, 30% eosinophils, 32% lymphocytes and 8% monocytes. However marked basophilia and eosinophilia at different stages of maturation detected by manual differential in PB smear revealed that automated blood cell counters wrong characterized the neoplastic basophilic cells with coarse and diffuse basophilic granules as neutrophils, as shown in Table 1.

**Table 1:** **Showing blood counts and chemistry profiles**

Laboratory Findings	At Presentation	1. month	3. month	5. month	7. month	7,5. month	8. month
**(×10** ^**9**^ **/L)**							
White Blood Cells	4.6	59.0	170.0	19.0	149.0	250.0	315.0
Basophil count	2.6	36.5	79.9	9.8	77.4		122.8
Eosinophil count	-	-	4.5	5.3	4.1		-
Mast cell count	-	-	28.9	3.0	23.8		78.7
Platelet	134.0	157	80.0	43.0	43.9		41.0
Hgb (g/dL)	5.7	8.9	7.1	8.9	8.9		7.2
***Manual Differential Counts (%)***							
Seg. Basophil	46	10	10		14		-
Basophil Band	8	12	5		2		1
Basophil Meta.	3	25	14		20		13
Basophil Myelo.	-	15	18		16		25
Promyelocyte	-	-	9		-		26
Eosinophilic series	29	6	27		28		-
Myeloblast	-	-	-		-		2
Mast cell	-	-	17		16		25
Tryptase (μg/L) ^†^	42.9		51.2				
Histamine (nmol/L)^††^	-		1.2				
IL-6 (pg/mL) ^†††^	-		38.5				
CRP (mg/dL)	-	85.6	225		30		55.9
LDH (U/L)	-	561	1221		-		5174
***Treatments***	Watch-wait	HU 1 g/L (×3 days)	leukaph. + HU 1.5 g/l (×3 days) + prednisolon 40 mg	İmatinib 300 mg/d + HU 0.5 g/d	leukaph. + Etoposide 50 mg	leukaph. + i.v. Cyclophos-phamide 1000 mg	EX

Magnification: 160 X 135 mm

^†^by F.E.I.A ImmunoCAP PHADIA, N < 11.4, treshold 11.4/l : 95th percentile (Cerba Lab. Paris France, Ref no: 887821335110 and Ref no 9197749), ^††^ by RIA (N < 10), ^†††^by Electrochemiluminescence ECLIA Roche (N < 7).

She was firstly transfused with 4 units of packed red blood cells (rbc) for severe anemia and maintenance therapy with HU was discontinued. Results of rutin laboratory studies with gastroscopy and colonoscopy failed to reveal gastrointestinal bleeding and hemolysis.

**Figure 1:**
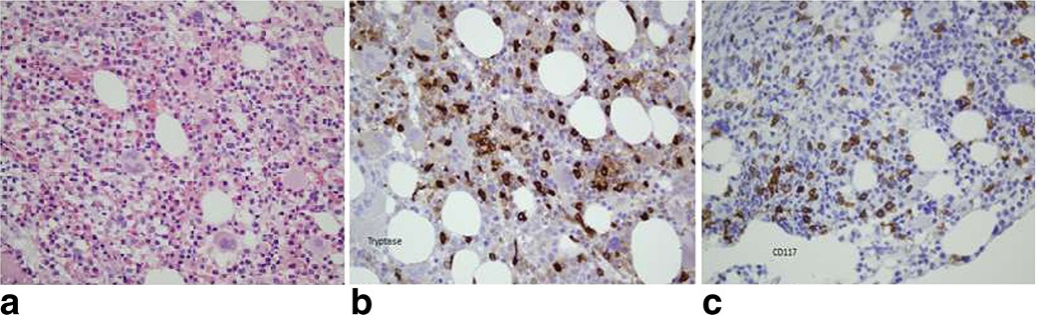
**Showing increased number of mast cells demonstrated by H&E stain in (a), immunohistochemical staining for tryptase in (b) and D117 in (c), respectively (×20).**

Bone marrow (BM) aspiration showed hypercellularity, basophilic hyperplasia, eosinophilia, increase in megakaryocytes and aggregates of mast cells. Suppressions in neutrophilic and in erythroid lineages induced by basophilic hyperplasia and MCs infiltration were seen. BM biopsy showed increase in megakaryocytes and eosinophils at different stages of maturation. Increased numbers of MCs in paratrabecular location were highlighted by immunohistochemical staining for CD117 and tryptase (Figure 1a, b and c). Increase in serum level of tryptase was shown in Table 1.

Diagnosis of SM occurring as second neoplasia concurrently with de novo CBL that relapsed after prolonged duration of hematologic remission was established. Her white blood cell (wbc) count except for the basophilia and eosinophilia was normal. The patient has been watched without treatment to see the recurrence of febrile episodes [7].

### Clinical course

Results of CBC and chemistry profiles during the clinical course were shown in Table 1.

*One month later* she was seen for symptoms of anemia, without fever. CBC showed increase in wbc and mast cell counts and decrease in Hgb. She was started on HU 1000 mg/day, but after 3 days, developed fever and her platelet count decreased to 63 × 10^9^/l. The patient stopped taking HU.

**Figure 2:**
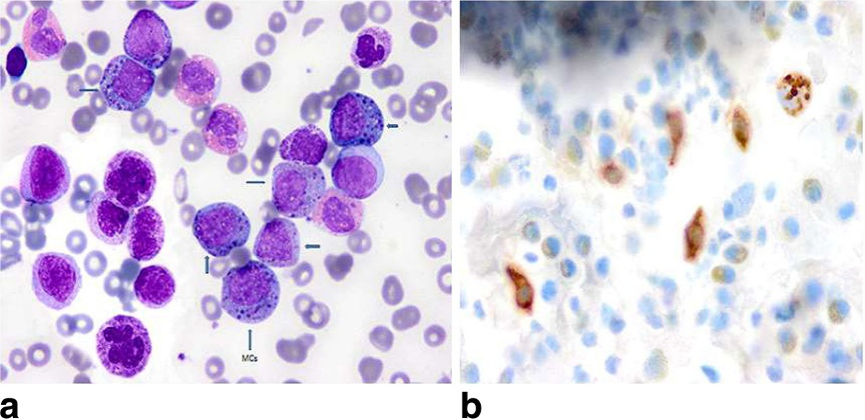
**Showing aggregates of mast cells containing mixed black and orange color round cytoplasmic granules and a giant segmented basophil in (a) (wright’s stain × 100), brown color round granular cytoplasmic staining demonstrated by tryptase immunohistochemical staining on PB smear representing mast cells in (b) (×100).**

**Figure 3:**
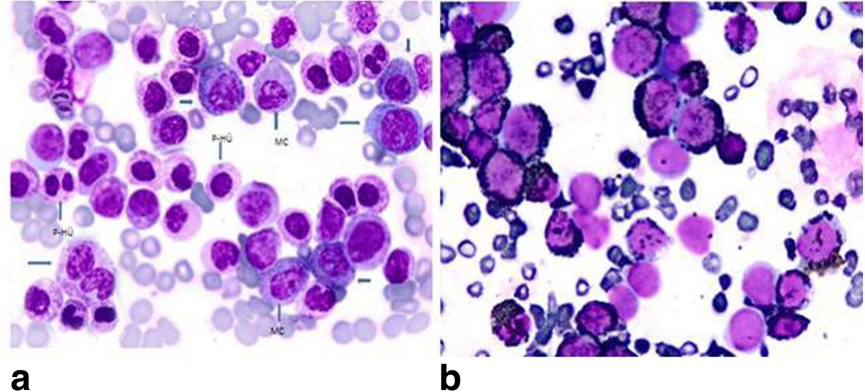
**Demonstrating basophils at various stages of maturation,giant binuclear basophilic metamyelocyte, aggregates of mast cells with mixed black and orange color round cytoplasmic granules and Pelger-Huёt anomalies in (a) (Wright’s stain, ×100). Showing diffuse granular staining by peroxidase stain in peroxidase positive basophils and absence of staining in aggregates of cells that representing myeloperoxidase negative mast cells in the BM in (b) (Peroxidase stain, ×100).**

About *2 months after (3. month of first presentation),* she presented with fever, abdominal pain, headache, weakness, diarrhea and was hospitalized. Repeat CBC revealed marked increase in wbc, basophil, eosinophil and mast cell counts, and decrease in Hgb. MCs showed uneven distribution with aggregates of 2 to 6 MCs on PB smear as they were seen in the BM smear. MCs have round nuclei, one or more nucleoli in immature forms and mixed black and orange color round cytoplasmic granules (Figure 2a). Her blood chemistry profiles were normal except for elevations in C-reactive protein (CRP) and lactate dehydrogenase (LDH). Laboratory studies performed during the febrile attack showed normal histamine, prominently increased IL-6 and elevated tryptase levels.Repeated BM aspiration showed hypercellularity, basophilic hyperplasia having diffuse, coarse basophilic granules and eosinophilia with presence of all stages of maturation of basophilic and eosinophilic lineages, including 2% segmented basophils, 7.5% basophlic bands, 17.5% basophilic metamyelocytes, 23.5% basophilic myelocytes, 1% segmented eosinophil, 4.5% eosinophilic bands, 8.5% eosinophilic myelocytes, 8% promyelocytes, 21.5% MCs. Marked suppression in neutrophilic lineage and moderate suppressions in erythroid and megakaryocytic lineages induced by basophilic hyperplasia and mast cell infiltrations were detected. The M:E ratio was 15.3:1. MCs exhibited uneven distribution with aggregates of 2 to 6 MCs throughout the marrow fields. Rare binuclear forms of MCs were also noted. Striking dysplasia manifested as giant segmented basophils, giant basophilic bands, giant binuclear basophilic metamyelocytes, frequent Pelger-Hüet anomaly (Figure 3a) and binuclear eosinophilic metamyelocytes were seen. A few giant hypersegmented megakaryocytes, hypogranular forms and rare megakaryoblasts were also observed.

Transformation to MCL from secondary SM occuring concurrently with accelerated phase of CBL relapse was detected 2 months after evolution to SM. The patient underwent 2 consecutive prophylactic leukapheresis to reduce to basophil count below 40 × 10^9^/l for the prevention of cytokine release from dead neoplastic cells induced by chemotherapy. She was transfused with 2 units of packed rbc and the treatment was resumed with increase in daily dose of HU to 1500 mg. Despite prophylactic leukapheresis, she developed febrile episode 3 days after chemotherapy. Her fever returned to normal with corticosteroid therapy, 40 mg/day, for 3 days, but she developed hematochezia due to decrease in Plt count to 28 × 10^9^/l. HU therapy was discontinued and she received supportive transfusion with plt packs and packed rbc.

The patient has received no treatment for about two months since she has required continuous supportive plt and rbc transfusions. Her wbc counts ranged betwen 4.5 × 10^9^/l and 18.3 × 10^9^/l and Plt counts ranged between 4 × 10^9^/l and 25 × 10^9^/l repectively during this period.

About *2 moths later (5. month of first presentation),* she was reevaluated for symptoms of anemia. CBC showed increases in basophil, eosinophil and mast cell counts and decrease in Hgb. Treatment with interferon alpha-2b (INFα-2b) was considered, but could not be administered because of persistent thrombocytopenia. She was treated with imatinib 300 mg/day combined with HU 500 mg/day during the ensuing month. She feld better, but decreases in Hgb to 7 g/dl and Plt count to 20 × 10^9^/l were noted. Combination therapy was stopped and she was given supportive plt and rbc transfusions.

Approximately, *7 weeks later (7. month)*, she developed fever, abdominal pain, fatigue and her spleen was palpable 4 cm below the left costal margin. CBC showed marked increases in wbc, basophil, eosinophil and mast cell counts and decrease in Hgb. The patient underwent 2 consecutive leukapheresis. Treatment with oral etoposide 50 mg/day was started, but on the thirth day of chemotherapy she developed fever and was hospitalized. Repeat CBC showed a wbc of 2.3 × 10^9^/l, Hgb 7.7 g/dl and Plt count 2 × 10^9^/l. Oral etoposide was discontinued because of thrombocytopenia (2 × 10^9^/l) and leukopenia (2.3 × 10^9^/l). She was treated with antibiotics and supportive transfusions. The patient was then followed for 2 weeks *(7.5 month)* without chemotherapy, but developed fever, abdominal pain and a rapid increase in wbc cunt to 250 × 10^9^/l was detected. She was started on leukapheresis and given i.v cyclophosphamide 1000 mg, but despite these treatments *2 weeks after (8.monts),* her wbc count continued to climb. Her biochemical and coagulation parameters were deteriorated rapidly. Repeat CBC revealed prominent increase in wbc, basophil and mast cell counts and decreases in Hgb and Plts. Coagulation profile revealed disseminated intravascular coagulation (DIC) during terminal phase. DIC was due to febrile neutropenia and infection that resulted in septic shock. Despite supportive managements with transfusions of fresh frozen plasmas, plt packs and packed rbc. DIC could not be controlled and she expired 4 days after development of DIC. The patient died 5 months after the diagnosis of MCL.

### Results of laboratory studies

Toluidine blue stain on PB and BM smears demonstrated diffuse granular metachromatic staining in the great majority of cells including MCs and basophils when transformation to MCL was detected. Peroxidase stain of BM smear showed diffuse granular staining in <60% of non-erythroid nucleated BM cells and peroxidase negative aggregates of cells (Figure 3b). Tryptase immunohistochemical staining was performed on PB smear by using Ventana Bench ultra automated staining apparatus and Ventana-Cell Marque-G3 mouse monoclonal antibody. Tryptase immunohistochemical staining of PB smear showed brown color round granular cytoplasmic staining in the aggregates of cells (Figure 2b).

Flow cytometric analysis of BM mononuclear cells (MNCs) showed that antigen expressions were positive for CD10 (dim), CD11c (dim), CD13, CD15, CD22 (dim), CD33, CD38, CD45, CD123, IgD receptor and CD117 and negative for HLA-DR,CD7 and CD71.

### Cytogenetic and molecular studies

Chromosome analysis was performed on 20 metaphases and abnormal karyotype was detected in six out of 20 metaphases. Cytogenetic analysis of BM cells revealed: 47,XX,der(6)t(6;?)(q25-27;?),der(17)t(17;?)(p13;?),+mar[6] / 46,XX[14].

Additional chromosomal materials were detected on the long arm of chromosome 6 and on the short arm of chromosome 17 respectively, in addition to a marker chromosme of unknown origin.

Sequence analysis performed on amplified PCR products of exons 9, 11,13 and 17 of c-kit gene revealed heterozygote substitution of C1650A > T (K550N) on exon 11 while wild type sequences were found in exons 9, 13 and 17 respectively. Molecular genetic studies performed by using LSI 4q 12 Tricolor Probe failed to reveal FIP1L1-PDGFRA fusion gene rearrangement.

## Discussion

CBL is a very rare type of leukemia, because seven patients with CBL appeared in the literature [4–7]. The two of the four cases reported as CBL by Pardanani et al. [4] could be categorized as SM-CBL according to the WHO major morphologic diagnostic criteria [3] as in the case described by Lahortiga et al. [5]. Because, an abnormal pattern of perivascular atypical mast cell infiltration were detected by tryptase immunohistochemical staining in these patients [4, 5]. Except for the cases reported by Lahortiga et al., Tang et al. and Cehreli et al. [5–7], flow cytometric analysis of antigen expressions required for the diagnosis of CBL were not present in four of the seven cases because this method was not available at that time [4]. Spesific stainings diagnostic for CBL was performed in only one [7] of the seven cases reported in the literature [4–7].

In our patient, MCs infiltration detected by immunohistochemical staining for tryptase and CD117 in the BM biopsy (Figure 1b, c) and elevation in tryptase level revealed evolution of SM occurring as a second neoplasia concurrently with de novo CBL relapse. Antigen expressions of CD10 (dim), CD11c (dim), CD13, CD15, CD22 (dim), CD33, CD38, CD45, CD123, IgD receptor and CD117 demonstrated by flow cytometric analysis of BM MNCs demonstrated infiltrations with basophilic cells [8–10] and MCs and supported evolution of SM occuring simultaneously with relapse of de novo CBL. Evolution of SM occurring as second neoplasia concurrently with de novo CBL that relapsed after prolonged duration of hematologic remission have not been seen in the literature [1–7].

Tansformation to MCL occurring from secondary SM with simultaneous development of accelerated phase of CBL was documented by the appearance of aggregates of MCs containing mixed black and orange color round cytoplasmic granules in both PB and BM smears (Figures 2a and 3a) in the reported patient. Marked dysplasia in neoplastic basophils and frequent Pelger-Hüet anomaly (Figure 3a) were the striking feature of the BM. Cytoplasmic granules of MCs may appear as either dark blue to purple and even blackish in color [11, 12]. Mast cells containing mixed black and orange color round cytoplasmic granules demonstrated by Hayhoe and Flemans in (figure 556) in the Colour Atlas of Haematological Cytology [12] and also shown in (Figures 2a and 3a) of our patient. Cytoplasmic granules may be seen on the nuclei of MCs.

Additionally, tryptase immunohistochemical staining of the PB smear in our patient showed round granular brown color cytoplasmic staining in the aggregates of cells confirmed that these cells demonstrated tryptase activity and represented MCs (Figure 2b). Because β-tryptase is a natural serine protease and is the most abundant mediator stored in the granules of mast cells [13]. This staining method could be utilized for the demonstration of undefined mast cells with atypical morphology on PB smear. MCs containing mixed black and orange color round cytoplasmic granules have not been described in patients with MCL appeared in literature [1–3].

Peroxidase stain showed diffuse granular staining in the majority of cells with presence of peroxidase negative aggregates of cells in BM and PB smears also disclosed transformation to MCL occurring simultaneously with accelerated phase of CBL. Because, mast cells do not contain peroxidase and appeared as peroxidase negative cells [14, 15], but basophils have basophil peroxidase [8] and stained with peroxidase, as seen in Figure 3b.

Diffuse granular metachromatic staining with toluidine blue stain on PB and BM smears was the supportive evidence revealing CBL relapse with evolution to MCL in our patient. Basophils and mast cells have electron-dense cytoplasmic granules and produce numerous inflammatory mediators such as histamine that are common to both cells and stain metachromatically with basic dyses, toluidine blue and alcian blue [11, 14]. Transformations to MCL occurring from secondary SM with simultaneous development of accelerated phase of CBL have not been described in patients with MCL reported in literature [1–7]. The accelerated phase of CBL in association with MCL, showed prominent dysplastic changes in basophilic, eosinophilic and megakaryocytic lineages of the BM with frequent Pelger-Hüet anomalies compared to the chronic phase of CBL [7].

Eosinophilia have been described in patients with CBL appeared in the literature [4–7], but after evolution of SM and transformation to MCL prominent eosinophilia, 5.3 × 0^9^/l in PB and BM were detected in our patient. Marked eosinophilia (>1.5 × 10^9^/l) was described in patients with SM-AHNMD, especially in 56% of SM-MPN and 39% of patients harbored FIP1L1-PDGFRA fusion gene [3]. FIP1L1-PDGFRA fusion gene rearrangement was found negative in our patient.

Our patient had weight loss and has had febrile episodes with abdominal pain when basophil counts increased >40.000 × 10^9^/l before or after chemotherapies with elevations in CRP and IL-6 levels. Although MCs and eosinophils were shown to produce IL-6 [16], but no febrile episodes were observed despite increases in MC count to 3 × 10^9^/l, eosinophil count to 5.3 × 10^9^/l because basophil count was 9.8 × 10^9^/l, less than 35 × 10^9^/l.

In addition, molecular studies performed during chronic phase of CBL by real time PCR in magnetic activated cell sorting (MACS) separated basophil fraction of BM MNCs demonstrated IL-6 gene expression in neoplastic basophils revealed that synthesis and release of IL-6 produced by neoplastic basophils in our patient [7].

MC activation symptoms included flushes, fever, malaise, diarrhea and tachycardia. Many patients were suffering from asthenia, severe weight loss and anorexia in 51 adult patients with MCL [2]. Elevations in CRP and IL-6 levels occurring with increases in basophil counts to high levels revealed that febrile episodes with abdominal pain seen in our patient were induced by increase in IL-6 levels released from neoplastic basophils [7, 17].

Literature review failed to demonstrate c-kit exon 11 K550N mutation and its effect on response to treatment and prognosis in patients with SM. In a phase III trial of imatinib mesylate for the treatment of advanced gastrointestinal stromal tumors studied by Cancer and Leukemia Group B and Southwest Oncology Group (CALGB 150105) revealed that the presence of *KIT* exon 11–mutant genotype (n:283) correlated with improved treatment outcome when compared with *KIT* exon 9–mutant (n:32) and wild-type (n:67) genotypes for objective response, complete response (CR) / partial response (PR) 71.7% *v* 44.4% (*P* .007) and 44.6% (*P* .0002), respectively [18].

Complete clinical and hematological remission were obtained by the treatment with imatinib in 3 out of five patients with SM showing eosinophilia and negative for c-kit D816V, but the other two who did not respond to treatment were the patients with the c-kit D816V positive. These results revealed that imatinib either inhibits the growth-promoting role of wild type c-kit, or targets as yet undefined a novel oncogenic kinase. [19, 20]. During transformation to MCL associated with accelerated phase of CBL our patient did not respond to combination treatment with imatinib and HU.

Responses to treatments during transformation to MCL occurring with simultaneous development of accelerated phase of CBL have been ineffective. Refractoriness to treatments was thought possibly due to development of resistant clones because cytogenetic studies showed an additional chromosomal material on the short arm of chromosome 17 where the p53 gene is located. The patient has had rapid and downhill course after transformation to MCL. Her biochemical and coagulation parameters were deteriorated rapidly and she developed DIC during the terminal phase. Despite supportive transfusions and antibiotics DIC could not be controlled and she expired 4 days after development of DIC. Transformation to MCL occurred 3 months after evolution of secondary SM and her survival was 5 months following the diagnosis of MCL. The median survival was 6 months in patients with MCL reported in the literature [2].

## Conclusion

MCL occurring as a second neoplasia in association with different MCs containing mixed orange and black color round cytoplasmic granules, increased IL-6 levels and accelerated phase of de novo CBL have not been reported in the literature [1–3]. Neoplastic basophils with diffuse and coarse basophilic granules as seen in Figure 2a in our patient possibly mimic neutrophils with toxic granules and cause wrong characterization of neoplastic basophils as neutrophils by the automated blood cell counters and misleaded the physicians in making differential diagnosis. Prophylactic leukapheresis should be considered as a palliative treatment to reduce leukocyte count and cytokine release from dead neoplastic cells induced by chemotherapies in patients with leukemias associated with high leukocyte counts and originating from the cells that are known to produce and release cytokine such as MCs, eosinophils [16] and neoplastic basophils [7]. In patients presented with recurrent attacks of fever and abdominal pain associated with progressive leukocytosis, eosinophilia and elevations in IL-6 and CRP levels, mastocytosis variants [21], chronic myeloproliferative disorders [22], Castleman’s disease [23] and possibility of underlying CBL [7] should be considered in the differential diagnosis. Manual differential count should be performed to rule out CBL.

### Consent section

The daughter of the patient has given her consent for the case report to be published.
